# Simulation of dosimetric consequences of intrafraction variation of tumor drift in lung cancer stereotactic body radiotherapy

**DOI:** 10.3389/fonc.2022.1010411

**Published:** 2022-12-08

**Authors:** Bin Han, Bian Wu, Fala Hu, Yangguang Ma, Haiyang Wang, Xinwei Han, Gang Liu, Yuexin Guo

**Affiliations:** ^1^ The First Affiliated Hospital of Zhengzhou University, Henan, Zhengzhou, China; ^2^ Cancer Center, Union Hospital, Huazhong University of Science and Technology, Tongji Medical College, Wuhan, China; ^3^ School of Mathematics and Statistics, Wuhan University, Hubei, Wuhan, China

**Keywords:** SBRT, 4D-CT, intrafraction, dose, tumor motion

## Abstract

**Objective:**

The purpose of this study was to investigate the target dose discrepancy caused by intrafraction variation during stereotactic body radiotherapy (SBRT) for lung cancer.

**Methods:**

Intensity-modulated radiation therapy (IMRT) plans were designed based on average computed tomography (AVG CT) utilizing the planning target volume (PTV) surrounding the 65% and 85% prescription isodoses in both phantom and patient cases. Variation was simulated by shifting the nominal plan isocenter along six directions from 0.5 mm to 4.5 mm with a 1-mm step size to produce a series of perturbed plans. The dose discrepancy between the initial plan and the perturbed plans was calculated as the percentage of the initial plan. Dose indices, including *ΔD_99_* for internal target volume (ITV) and gross tumor volume (GTV), were adopted as endpoint samples. The mean dose discrepancy was calculated under the 3-dimensional space distribution.

**Results:**

We found that motion can lead to serious dose degradation of the target and ITV in lung SBRT, especially during SBRT with PTV surrounding the lower isodose line. Lower isodose line may lead to larger dose discrepancy, while make steeper dose fall-off gradient. This phenomenon was compromised when 3-dimensional space distribution was considered.

**Discussion:**

This result may provide a prospective reference for target dose degradation due to motion during lung SBRT treatment.

## Introduction

It is known to be different from conventional radiotherapy, stereotactic body radiotherapy (SBRT) with altered dose-fraction regimens is increasingly being utilized in the management of early-stage lung cancer (1, 2). SBRT has demonstrated significant improvements in local control and overall survival (3). In lung SBRT, a high dose is delivered to the tumor with a highly conformal beam arrangement, with minimal dose delivered to critical nearby normal tissues (4). However, respiratory-induced target motion may lead to tumor geometric uncertainty, thereby reducing local control and increasing the chance of off-target radiation delivery to nearby organs (5). Therefore, respiratory motion must be managed and controlled during both simulation and treatment.

Four-dimensional computed tomography (4DCT) has been already a standard radio imaging tool, as it is capable of detecting motion and deformation of the entire tumor during a breathing period (6). Utilizing 4DCT in planning target volume(PTV) design is currently the most popular method to compensate for respiratory-induced target motion in treatment planning (7). The target manifested on the 4DCT images is assumed to represent the target motion during treatment, although respiratory patterns may change with time and intrafraction variation (IFV) can occur (8, 9). Due to tight PTV margins and steep dose gradients in SBRT, such significant geographic errors can result in unnecessary irradiation of healthy tissues and compromise dosage to the target. Due to the target deformation, amplitude of tumor movement, and imaging artifact in the 4DCT, the dosimetric consequences of mobile target quantitatively using the patient dataset directly during lung SBRT is inconvenient. It is suggested that using a digital lung cancer phantom as an alternative in previous studies (10, 11).

Many methods have been used to study the quantitative effects of tumor movement on the target area, while there is no standard method until now (12, 13). Target and clinical factors influencing the volume and dose derived from 4DCT and Cone beam CT was evaluation of lung cancer (14). Many researchers have been trying to find better methods to study the influencing of tumor motion by all kind of simulations (15, 16). The tumor motion is deformable whereas the isocenter location change is rigid when it comes to the treatment planning calculation of targets as well as critical structures. Therefore, a suitable simulation method has been looking for by many researchers. Considering that the simulation must be realistic and convenient, both the simulations of phantom and the motion are virtual which is meaningful for relevant research.

The goal of this study was to investigate the target dosimetric effect caused by variation during SBRT for lung cancer. For this purpose, a series of lung motion phantoms with different tumor sizes and motions were created through computer simulation to perform SBRT to model target dosimetric sequence caused by infraction motion. Then several clinical lung cancer patients received SBRT were retrospective to validate.

## Methods and materials

### Digital lung cancer phantoms

**Figure 1:**
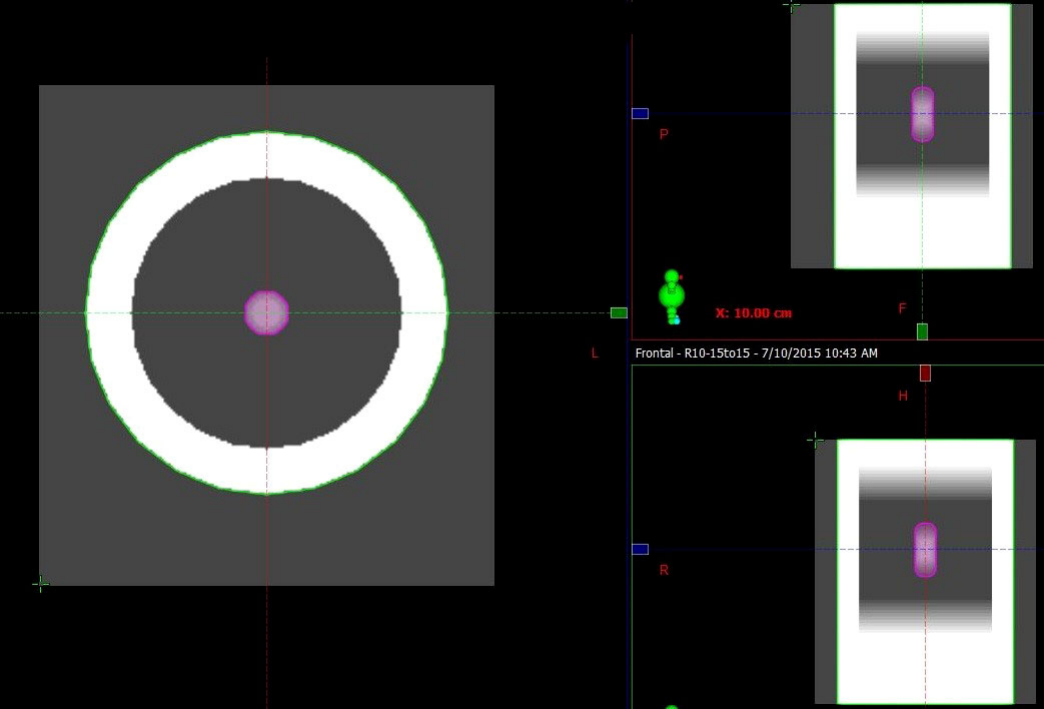
Example of AVG CT of a respiratory digital lung cancer phantom. The ITV contoured in the MIP (pink contour), chest wall (green contour) and lung (between the tumor and chest wall) are shown.

The mechanics of respiration (induction of an expansion–contraction motion) are significant in the superior inferior (SI) direction for lung cancer patients. Peak-to-peak amplitudes have been reported to range from 0 to 3 cm (17). A series of digital lung cancer phantoms similar to those utilized in previous studies were generated through an in-house program to simulate tumor sizes of 2, 3, and 4 cm and rigid motion with an amplitude of 1, 2, and 3 cm (18). A total of nine cases (tumor size vs. motion amplitude) were included in the simulation, where eleven phases spanned a half-cycle of respiration according to the amplitude, with 0% phase representing the tumor in peak position and 60% phase representing the tumor in the valley position. The CT number of the chest wall, lung and tumor was assigned as 0, -720 and 0 Hounsfield units (HUs), respectively. GTV contours were delineated *via* the threshold according to the HU of the tumor utilizing Velocity software (Varian Medical System version 3.1). Maximal intensity projection CT (MIP CT) and AVG CT were generated by maximizing and averaging the voxel intensities of all eleven phases, respectively (the AVG is shown in **Figure 1** as an example). An ITV encompassing the GTVs was generated on the simulated 4DCT image within a single breathing cycle.

### SBRT planning

The static intensity-modulated radiation therapy (IMRT) plan was created using 9 equal angle beams to the PTV center in Eclipse TPS with 6-MV photon flattening filter beams. A 5-mm isotropic expansion around the ITV produced the PTV. The treatment prescription dose was 6000 cGy to PTV in 5 fractions. To achieve the prescription dose, the planned treatment dose was calculated based on the average AVG CT image using the Acuros XB (AXB) algorithm (AXB), and a prescription dose of 6000 cGy was applied to the isodose line (65% or 85%) that covered at least 95% of the PTV (19). The average total monitor units (MUs) was 1005 (range 920~1104).

### Patient study

Three non-small cell lung cancer cases with varying tumor location, size, and magnitude of motion were enrolled as clinical cases for validation purposes. All 4DCT image data acquisition was performed using multislice helical CT (Philips Medical Systems, Cleveland, OH, USA). Each 4DCT with ten phases was generated with 3-mm thickness utilizing the phase-based method (20).

The GTV was delineated on each phase image with the CT pulmonary window utilizing Velocity software (Varian Medical System, version 3.1). The ITV was defined as the union of all GTV phases on the simulated 4DCT image within a single breathing cycle. The static IMRT plan with 8-10 coplanar fields was designed for each patient using Eclipse with 6-MV photon flattening filter beams. An isotropic 5-mm margin was applied for ITV-to-PTV expansion. At least 95% PTV was covered by the prescription dose. The characteristics of the targets and associated plan parameters are given in **Table 1**. The average total monitor units (MUs) is 2160 (range 2068~2314).

**Table 1:** Tumor characteristics and dose differences for the ITV and GTV analyses of the three patients.

Patient	Location	Diameter	Amplitude	Prescription Isodose	Regimen	ITV		GTV	
No.		(cm)	(cm)			$\bar{\Delta D_{99}}$ (%)	$\bar{\Delta D_{95}}$ (%)	$\bar{\Delta D_{99}}$ (%)	$\bar{\Delta D_{95}}$ (%)
1	RLL	2.9	0.5	76%	60 Gy/5 f	-1.7	-0.5	-1.4	-0.4
2	LLL	1.9	1	83%	60 Gy/5 f	-0.9	-0.3	-1.2	-0.4
3	RUL	2.2	3	75%	60 Gy/5 f	-1.5	-0.4	-2.0	-0.5

LLL, left lower lobe; RLL, right lower lobe; RUL, right upper lobe.

This study was approved by the Institutional Review Board at the Tongji Medical College of Huazhong University of Science and Technology. All methods were performed in accordance with the relevant guidelines and regulations. Written informed consent was obtained from all authors and participants.

### Variation simulation

variance was defined as the displacement of the tumor location recorded in the final post-treatment cone beam computed tomography (CBCT), which was simulated by discretely shifting the planned isocenter along the superior, inferior, left, right, anterior and posterior directions to a series of static position samples (21). In this study, the isocenter position shifts were assigned as 0.5 mm, 1.5 mm, 2.5 mm, 3.5 mm and 4.5 mm. Cases in which the isocenter was moved simulated the situation when all beams were incorrectly aimed in the same direction during treatment due to mean target position variation, resulting in a perturbed plan.

### Variation in a clinical case

According to the workflow described previously, localization of tumors was implemented for three enrolled patients (22). A tolerance of 3 mm was followed through an online image registration process. Precorrection CBCT images were reconstructed and manual soft tissue (tumor) matching was performed with the tolerance for repositioning in any of the three orthogonal directions. Post-correction CBCT images were obtained and measurements of residual error were performed in the superior-inferior (SI), anterior–posterior (AP), and medial–lateral (ML) dimensions. This procedure was continued to determine the residual error within the tolerance in all dimensions. At the end of treatment, a final CBCT scan was acquired to assess target motion. A three-dimensional vector was calculated from target motion with the formula $\sqrt{x^{2}+y^{2}+z^{2}}$ , where *x*, *y*, and *z* correspond to displacements in the ML, AP, and SI directions, respectively. Target dose variation was assessed with the treatment fraction under the max 3D vector for each patient.

### Evaluation

To calculate the total dose for a moving target, it is necessary to trace the voxel motion trajectory during the respiratory cycle. The grayscale image intensity-based deformable image registration method was utilized to determine the voxel-by-voxel displacement vector, which linking the geometric coordinates between the reference phase image and other phase images for patient cases (21), while only rigid registration was performed for phantom cases. The End-exhalation (EE) phase was set as the reference phase in this study (22). A simplified method for 4D dose accumulation was implemented by replacing each static phase dose by the same AVG CT dose distribution to save time in this study (23). The dose distribution calculated using the designing CT set is referred to as the initial dose distribution. The dose distribution for a series of perturbed plans was also calculated.

The largest percentage dose level *D_x_* denotes the *x*% volume of a structure, and Δ*D_x_* denotes the percentage relative to the initial planned value to quantify the deviation. The dose indices, including *ΔD_99_*, generated between the initial plan and the series of perturbed plans were acquired as endpoint samples.

Variation has been quantified in previous studies. For example, Li et al. evaluated variation in 133 patients undergoing lung SBRT with CBCT-based correction and found 1 standard deviation (1 *δ* of variation to be 1.5, 1.5 and 1.2 mm in the SI, AP and ML dimension acquired post-treatment, respectively (24), which were independent in 3 directions and had a normal distribution. The 3-dimensional space from 0 to 3· *δ* was assigned as a series of twelve spaced samples each covering a range of 0.25· *δ* . The corresponding probability of each space to be occupied was calculated and denoted as *P_i_*, where *i*=1,2,…12 to cover the entire 3-dimensional space. The probability of partitioning out of 3· *δ* was included in *P_12_*. Under spherical coordinates, the probability that the length of the vector (*x, y, z*) is smaller than *i*·0.25·*δ* is given as (25)


(1) $$Q(i)=\int_{0}^{i\cdot 0.25\cdot \delta }\frac{r^{2}}{\sqrt{\frac{\pi }{2}}\cdot \delta^{3}}\cdot e^{-\frac{r^{2}}{2\cdot \delta^{2}}}dr$$

therefore *P*_*i*_=*Q*(*i*)−*Q*(*i*−1) , where *Q*(12)=*Q*(+*∞*) . The isocenter position shift was defined from 0.125· *δ*_*x*_ (.represents the component in the *x* direction) to 2.875· *δ*_*x*_ with 0.25· *δ*_*x*_ steps along the patients’ left and right directions. A similar method was implemented along the patients’ superior, inferior, anterior and posterior directions. The corresponding *ΔD_99_* and *ΔD_95_* were calculated using endpoint samples acquired with the determined isocenter shift described above through linear interpolation.

The symbol $\Delta D_{x}^{\overset{\vec{r}}{\mathrm{ij}}}$ denotes $\Delta D_{x}$ between the initial plan and the perturbed plan with the isocenter shifting distance as $\vec{r_{i}}$ along the patients’ *j* direction, where $\vec{r_{i}}\in [0.125\cdot \delta ,\;0.375\cdot \delta \ldots ,\;2.875\cdot \delta ]$ , *i*=1, 2…,12, and *j*=1, 2…6, representing the direction along the patients’ anterior, posterior, right, left, superior, and inferior directions, respectively.

The average $\Delta D_{x}$ determined by the isocenter shifting distance $\vec{r_{i}}$ was denoted as:


(2) $$\Delta D_{x}^{\vec{r_{i}}}=1/6\sum_{j=1}^{6}\Delta D_{x}^{\overset{\vec{r}}{i,j}}$$

The mean variance of the target dose during treatment was defined as:


(3) $$\bar{\Delta D_{x}}=\sum_{i=1}^{12}p_{i}\times \Delta D_{x}^{\overset{\vec{r}}{i}}$$

and was calculated to analyze all cases.

## Results

**Table 2** lists all *P_i_* values. Under normal distribution, the space samples are homogeneous, but each space sample has heterogeneous probability. The largest probability was 14.6% and the smallest was only 0.4%.

**Table 2:** Probability of *P_i_*.

Index	*P_1_*	*P_2_*	*P_3_*	*P_4_*	*P_5_*	*P_6_*	*P_7_*	*P_8_*	*P_9_*	*P_10_*	*P_11_*	*P_12_*
Probability (%)	0.4	2.7	6.4	10.4	13.3	14.6	14.0	12.1	9.4	6.7	4.4	5.6

### Phantom study

**Figure 2:**
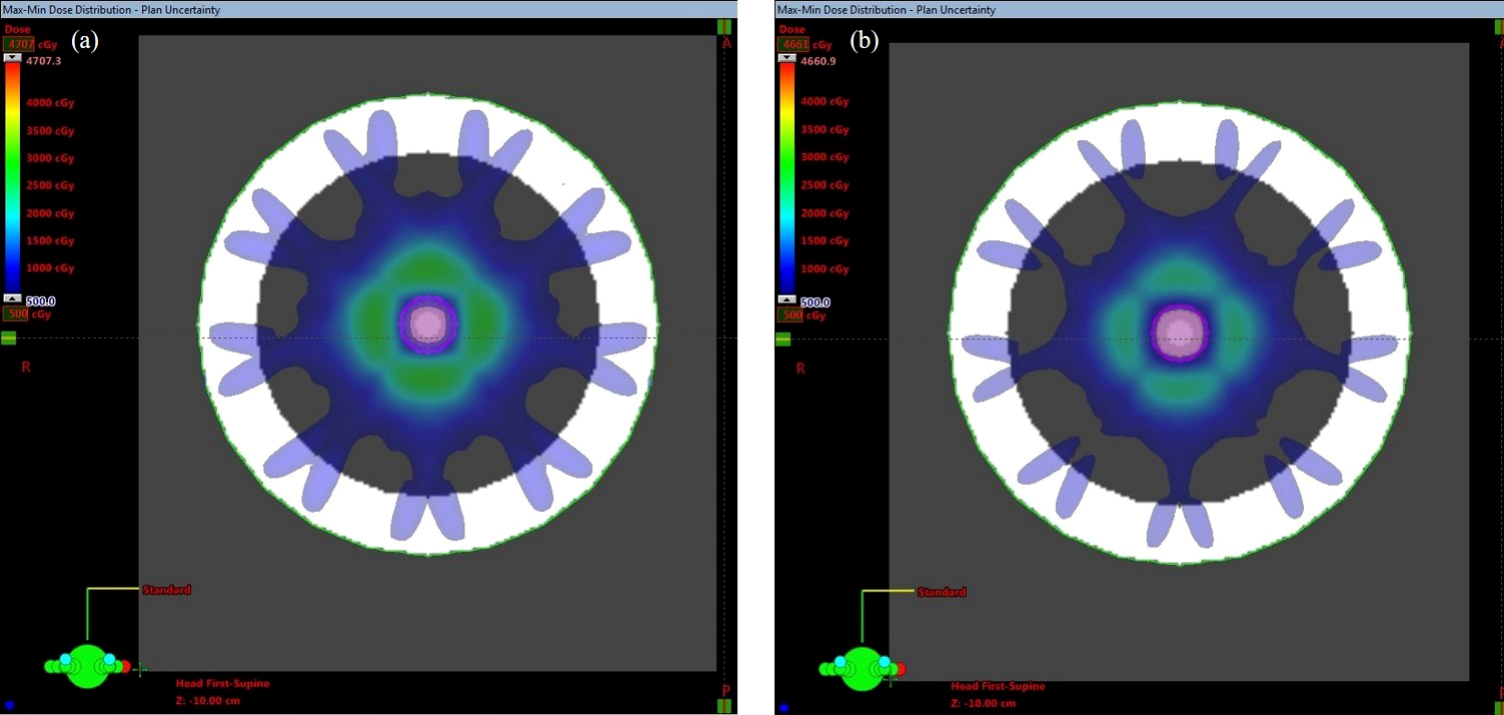
Max-min dose of the PTV covered by **(A)** 65% and **(B)** 85% of the isodose line. The pink contour represents the ITV.

The max-min dose can reveal where the biggest difference in dose may occur if all plan uncertainty doses are considered. This dose is a derived dose difference calculated based on the dose from the original plan and all plan uncertainty doses. **Figure 2** shows the max-min dose. It is clear that the dose difference appeared around the ITV, especially at the edge of the ITV. Attention must be played to the area in the ITV; the dose difference disappeared in the middle of the ITV under the 500 cGy threshold, while the dose difference was still observed in the region around the ITV edge in the plan with the dose level of 65% compared to the plan with the dose level of 85% (larger region in the ITV).

**Figure 3:**
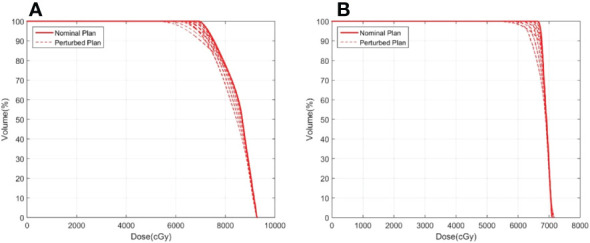
Example of ITV DVHs for plans with prescription isodoses of **(A)** 65% and **(B)** 85%. The solid line represents the nominal plan and the dashed line represents the perturbed plan.

**Figure 3** shows an example of the DVH analysis for the digital phantom study. The series of perturbed plans was produced by shifting the isocenter from 0.5 mm to 4.5 mm in the nominal plan along the patient’s anterior, posterior, right, left, superior, and inferior directions. A wider variation in the DVHs of the ITV was observed for the plan with a dose level of 65% than for the plan with a dose level of 85%. The dose discrepancy in the ITV was degraded with an increase in the isocenter shift, especially at doses less than *D_60_*. A similar phenomenon was observed in other cases, implying that higher prescription isodose lines would be selected during planning in clinical practice. For all endpoints examined, *ΔD_99_* varied between 0.0% to -7.7%for ITV vs. -0.2% to -11.5% for GTV. 19.9% (1 *δ* ) of *ΔD_99_* was greater than -1.4% for ITV and was -3.1% for GTV in. 73.9% (2 *δ* ) of *ΔD_99_* for ITV was larger than -4.2%, and *ΔD_99_* for GTV was larger than -6.9%. The results do not demonstrate a relationship between the endpoints and target size or target motion amplitude, warranting further research in the future. However, the results provide a prospective reference for target dose degradation due to motion during lung SBRT treatment by calculating the probability distribution.

Motion affects the actual delivered dose to the target. However, when averaged over the distribution of variance 3-dimensional space, $\bar{\Delta D_{99}}$ degradation only larger than -2.9% for ITV vs. -5.0% for GTV.

### Patient study

**Figure 4:**
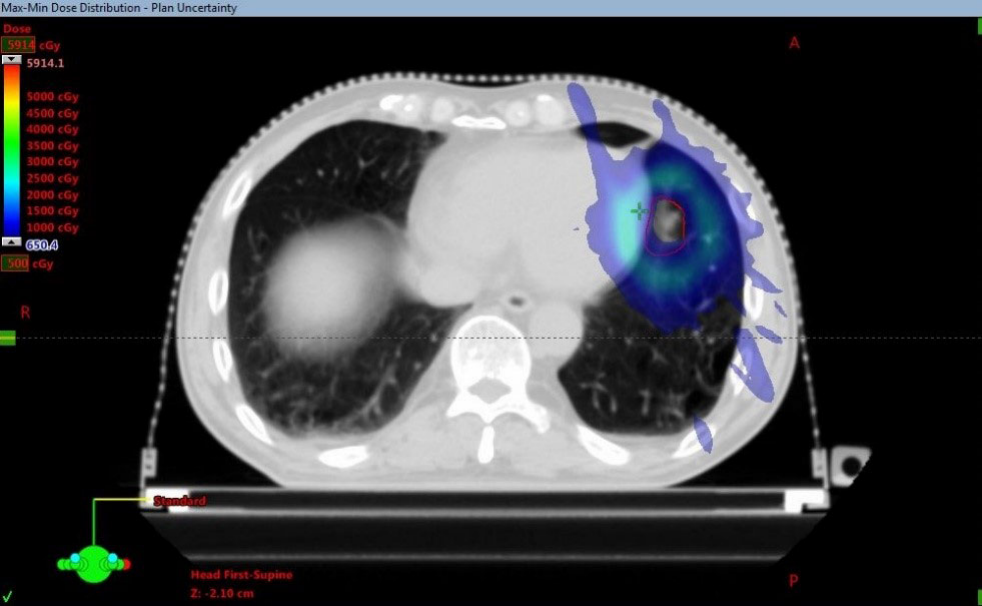
Max-min dose examples from patient 2. The red contour represents the ITV.

The tumor characteristics of the three patients are listed in **Table 1**. The tumor was located in the right lobe for patient 1, the left lobe for patient 2, and the right upper lobe for patient 3; the amplitude of tumor motion was 0.5 cm, 1 cm, and 3 cm, respectively. The dose discrepancies, *ΔD*_99_ , for the ITV and GTV are listed in **Table 3**. The *ΔD_99_* varied from 0.0% to -4.9% for ITV vs. 0.0% to -5.6% for GTV. However, under a 3-dimensional distribution, the $\bar{\Delta D_{99}}$ ecreased to larger than -1.7% for ITV vs. -2.0% for GTV (**Table 1**). **Figure 4** shows the max-min dose for patient 2 as an example. It is also clear that the dose difference appeared around the ITV, especially at the edge of the ITV. Although the complicated deformable image was obtained from a real patient, the results are consistent with those from the phantom study. Dose degradation is greater for the plan with prescription isodose lines of 73% and 75% than for the plan with a prescription isodose line of 83%. The results demonstrated that the plan with the 83% prescription isodose line is more robust, which is consistent with the typical prescription isodose line of approximately 80% in the radiation therapy oncology group (RTOG) 0813. However, the judgment should be further researched based on the data provided in this study since the real patient shows the target size, target motion amplitude and target shape deformation.

**Table 3:** Dose difference of ITV and GTV variation along with the isocenter shift.

Patient No.	ROI	Index	Isocenter Shift ( *δ*)											
			0.125	0.375	0.625	0.875	1.125	1.375	1.625	1.875	2.125	2.375	2.625	2.875
1	ITV	ΔD_99_(%)	0.0	-0.2	-0.5	-0.7	-1.1	-1.5	-2.0	-2.5	-3.2	-3.8	-4.3	-4.9
		ΔD_95_(%)	0.0	-0.1	-0.2	-0.4	-0.6	-0.8	-1.2	-1.5	-2.0	-2.5	-2.8	-3.4
	GTV	ΔD_99_(%)	-0.1	-0.3	-0.5	-0.6	-0.9	-1.2	-1.6	-2.1	-2.6	-3.2	-3.8	-4.5
		ΔD_95_(%)	-0.2	-0.4	-0.5	-0.6	-0.7	-0.9	-1.2	-1.5	-1.9	-2.2	-2.5	-3.1
2	ITV	ΔD_99_(%)	0.0	0.0	-0.2	-0.3	-0.5	-0.8	-1.1	-1.4	-1.8	-2.3	-2.7	-3.2
		ΔD_95_(%)	0.0	-0.1	-0.2	-0.3	-0.4	-0.6	-0.8	-1.0	-1.3	-1.6	-1.8	-2.2
	GTV	ΔD_99_(%)	-0.2	-0.5	-0.7	-0.8	-1.0	-1.2	-1.5	-1.8	-2.2	-2.5	-2.8	-3.2
		ΔD_95_(%)	-0.2	-0.5	-0.6	-0.6	-0.8	-0.9	-1.1	-1.3	-1.5	-1.8	-2.0	-2.3
3	ITV	ΔD_99_(%)	0.0	-0.1	-0.4	-0.6	-1.0	-1.3	-1.8	-2.3	-2.9	-3.5	-4.1	-4.8
		ΔD_95_(%)	0.0	0.0	-0.2	-0.4	-0.6	-0.8	-1.2	-1.5	-1.9	-2.3	-2.6	-3.1
	GTV	ΔD_99_(%)	-0.1	-0.3	-0.6	-0.9	-1.3	-1.8	-2.4	-2.9	-3.6	-4.3	-4.9	-5.6
		ΔD_95_(%)	0.0	-0.3	-0.5	-0.7	-0.9	-1.1	-1.5	-1.8	-2.2	-2.7	-3.1	-3.7

The tumor motion values with the max 3D vectors for patient 1, patient 2 and patient 3 are (2.9, -2.0, -0.9) (unit: mm), (-1.5, -0.4, 3.5), and (-2.0, 0.4, 3.6) respectively. The corresponding target degradation values are *ΔD*_99_ for ITV: -2.6% vs. *ΔD*_99_ ( *ΔD*_95_ ) for GTV: -2.5%, *ΔD*_99_ for ITV: -1.6% vs. *ΔD*_99_ for GTV: -2.2%and *ΔD*_99_ for ITV: -2.2% (-1.6%) vs. *ΔD*_99_ for GTV: -2.8%, respectively.

## Discussion

Digital phantoms generated *via* computer simulation as well as patient cases were analyzed to investigate the target dose discrepancy caused by variation during SBRT for lung cancer. The results suggest that the variance plays an important role in target dose degradation; *ΔD_99_* for the ITV and GTV reached -7.7% and -11.5%, respectively, even in the phantom study (26). Additionally, the dose distribution in the ITV and GTV was greatly affected by a steeper dose gradient regarding the 3-dimensional space distribution. $\bar{\Delta D_{99}}$ degraded to larger than -2.9% for ITV *vs*. -5.0% for GTV in phantom cases and to -1.7% (-0.5%) for ITV *vs*. -2.0% for GTV in patient cases.

**Figure 5:**
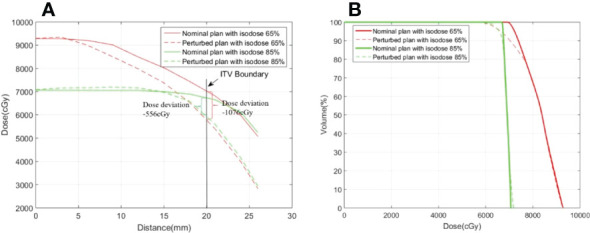
Examples of **(A)** a dose profile and **(B)** a dose volume histogram in the ITV for the nominal plan and the perturbed plan with prescription isodoses of 65% and 85%. The dose profiles cover the range from the isocenter to the ITV boundary along the SI direction.

This study demonstrates that prescription isodose is an important factor for target dose variation, especially the PTV covered by the lower isodose line. **Figure 5A** displays an example from the phantom study of the dose profile from the isocenter to the ITV boundary for the nominal plan and a perturbed plan with a prescription isodose of 65% and 85%, respectively, where the perturbed plan was produced through shifting the isocenter by 4.5 mm relative to the nominal plan along the patient’s SI direction. A sharper dose profile was observed in the nominal plan with a lower dose level. Dose degradation between the nominal plan and the perturbed plan in ITV produced with the same isocenter shifting was more sensitive, especially for the voxel in the ITV boundary. Thus, the nominal dose profile can be predicted using the following equation (27):


(4) $$\Delta D(v)\approx \mu^{T}(x_{0})\cdot \bar{\nabla }d[x_{0},x_{0}+u(x_{0})]$$

where *d*(*x*_0_) represents the subvolume at position *x_0_*, *ΔD*(*v*) represents the dose deviation of subvolume *v*, *μ*(*x*_0_) is the temporal displacement or variance of the subvolume, and $\bar{\nabla }d[x_{0},x_{0}+u(x_{0})]$ is the mean dose gradient within the interval [*x*_0_,*x*_0_+*u*(*x*_0_)] . The dose deviation for the voxel in the ITV boundary was -1076 cGy (actual) *vs*. -1120 cGy (predicted) with the prescription isodose of 65%, and -556 cGy (actual) *vs*. -615 cGy (predicted) with the prescription isodose of 85%. However, as shown in the profile of the nominal plan displayed in **Figure 5A**, the corresponding mean dose gradient $\bar{\nabla }d[x_{0},x_{0}+u(x_{0})]$ was -249 cGy/mm vs. -137 cGy/mm, respectively, where *x_0 =_* 20*_ mm_* and *u(x_0_)*=4.5 mm. Greater dose degradation at lower dose levels relative to higher dose levels was also observed in the DVH displayed in **Figure 5B**, especially for the endpoint with the lower dose. In fact, *ΔD_99_* was -13.4% and -7.2% (-2.6%) for plans with prescription isodoses of 65% and 85%, respectively. Therefore, during SBRT treatment, due to respiratory motion, a lower dose level (i.e., a steeper dose gradient) may result in more dose variance.

Li et al. also reported that 1 standard deviation (1 *δ* ) of variation was 1.5, 1.4 and 1.1 mm in the SI, AP and ML dimension prior to beam delivery of the non-coplanar beams, respectively, which is less than the one (1.5,1.5,1.2 mm); acquired at the end of treatment. Meanwhile, the lower treatment delivery time was cost in the case prior to beam delivery of the non-coplanar beams (noted case_mid_) compared to the one acquired in the case end of treatment (noted case_end_). ITV D99 degradation was slightly improved in the case_mid_ compared to the one acquired in case_end_. More specially, for phantom simulation, 19.9%(1 *δ* ) *of ITV ΔD_99_* could be improved from >-1.4% (case_end_) to > -1.3%(case_mid_) and *GTV ΔD_99_* could be improved from > -3.1(case_end_) to > -2.9%(case_mid_),respectively. 73.9% (2 *δ*) of ITV *ΔD_99_* was improved to >-4.0%(case_mid_) vs -4.2(case_end_), and GTV *ΔD_99_* was improved to -6.6% in case_mid_ vs >-6.9% in case_end_. It indicated that treatment delivery time reduction or treatment efficient improvement is an effective strategy to mitigate intra-fraction variation. Furthermore, the VMAT and dose rate with flattening filter free (FFF) model were demonstrated to reduce treatment delivery time compared to the conventional IMRT in lung SBRT in previous study (28, 29). Therefore, it can be deducted that the approach of using VMAT or high dose rate with FFF model would be available to compromise the dosimetric consequences leaded by intra-fraction variation in lung SBRT.

Our results show that the dose difference in the ITV differs from that in the GTV. Thus, the dose distribution in the ITV cannot accurately predict the actual target dose in lung cancer SBRT, which is consistent with previous studies (5).

There are some limitations to this study. To access the accumulative dose for the mobile target, each phase dose was calculated, mapped to the reference phase, and summed. Therefore, to acquire an accumulative dose with perturbation, each phase dose was calculated under isocenter shift samples. The time consumed included 30 samples * 11 phase * 2 dose levels * 3 diameters * 3 motion amplitudes (5940 calculations). To save time, a simplified method for 4D dose accumulation was implemented by replacing each static phase dose by the same AVG-CT dose distribution. This method significantly reduced the time by fractions of 1/11 and 1/10 for the phantom case and the patient case, respectively. The target dose difference between the two methods was investigated so that the endpoint indices, including those for *D_min_*, *D_99_* and *D_1_*, were less than 2% (18), which was considered acceptable. The three-dimensional space was divided into twelve spaced samples from 0 to 3· , with each sample covering a range of 0.25· *δ* Although the endpoints were acquired by shifting the isocenter along six directions, the sample endpoints were not sufficiently matched, which may have resulted in an inaccurate calculation of the probability of each space sample, *P_i_*. Therefore, more samples are required to divide the three-dimensional space in order to accurately determine the probability of each space sample; this will be implemented in the future.

As far as we know, there are some ways to reduce such variations and thereby minimize the dosimetric degradation of the target in lung cancer for SBRT. A novel 4D robust planning strategy to compensate for such heterogeneity respiratory motion has been explored in our earlier study (11). Special postural fixation methods can also reduce the impact of exercise, such as Body-FIX system and Abdominal pressure plate technique. At the same time, a method that breath hold technique combined with fast CBCTs can limit the motion of tumor efficiently.

Another limitation was that only three tumor sizes were included in the digital phantom study, which covered tumor volume in actual SBRT lung cancer patients insufficiently. We will investigate the target volume effects in SBRT for lung cancer in depth in the future.

## Conclusions

In general, the results of our study have shown target dose simulation to be an appropriate tool for a better understanding of the influence of intra-fraction variation of tumor drift in lung cancer stereotactic body radiotherapy. Motion of tumor, surrounded by tissue of lower density, leads to a variant dose distribution during the tumor drift. The approach of using VMAT or flattening filter free (FFF) model would be an available to compromise the dosimetric consequences.

## Data availability statement

The original contributions presented in the study are included in the article/supplementary material. Further inquiries can be directed to the corresponding authors.

## Ethics statement

The studies involving human participants were reviewed and approved by Institutional Review Board at the Tongji Medical College of Huazhong University of Science and Technology. The patients/participants provided their written informed consent to participate in this study.

## Author contributions

Conception and design: BH, BW, FH, GL. Acquisition of the data: BH, YM, HW. Analysis of the data: BH, FH. Writing, review and/or revision of the manuscript: BH, XH, GL and YG. All authors contributed to the article and approved the submitted version.
